# A Golden-Ratio Ladder and a Delocalisation-Saturated Participation Bridge for the Hydrogen-Bond Network of Liquid Water

**DOI:** 10.3390/molecules31152701

**Published:** 2026-08-03

**Authors:** Jonathan Washburn, Elshad Allahyarov

**Affiliations:** 1 Recognition Science Research Institute, Austin, TX 78701, USA; jon@recognitionphysics.org; 2 Institut für Theoretische Physik II: Weiche Materie, Heinrich-Heine Universität Düsseldorf, Universitätsstraße 1, 40225 Düsseldorf, Germany; 3 Theoretical Department, Joint Institute for High Temperatures, Russian Academy of Sciences (IVTAN), 13/19 Izhorskaya Street, Moscow 125412, Russia; 4 Physics Department, Case Western Reserve University, Cleveland, OH 44106, USA

**Keywords:** liquid water, hydrogen bonding, H-bond network topology, Bethe lattice, golden-ratio ladder, molecular dynamics, TIP4P/Ew, instantaneous normal modes, far-infrared/THz spectroscopy

## Abstract

We organize the ultrafast hydrogen-bond timescales and far-infrared/terahertz band centres of liquid water on a single golden-ratio ladder, anchored to the 80 fs H-bond contraction time and indexed by a loop-free tetrahedral Bethe sequence. Both data sets fall inside the rung windows set by their shell index, placing them on one spacing from a single time anchor. A separate empirical energy anchor gives a calibrated map of the freezing and density maximum. A TIP4P/Ew analysis shows that participation is delocalisation-saturated rather than linear in shell size, and we fit a corresponding saturating law. The low-frequency intermolecular modes are delocalised over the network, with coherent shell-breathing extending over about one coordination shell, a result stable with temperature. The ladder placements are therefore an organizing construction with explicit calibrations. A statistical assessment (a scan over alternative logarithmic bases and a null-model randomisation) confirms that the bracketing does not statistically single out the golden ratio, so the physical conclusions rest on the measured participation law, not the ladder. Together these establish a calibrated bridge between hydrogen-bond-network topology and vibrational participation, with the ladder supplying the rung spacing and the molecular dynamics the participation law that populates it.

## 1. Introduction

The structure and dynamics of liquid water have been studied since the 1930s tetrahedral-network proposals of Bernal–Fowler [1] and Pauling [2], through Stillinger [3] to modern diffraction [4,5,6] and far-infrared/THz spectroscopy [7,8,9,10]. Liquid water is locally close to tetrahedral but its H-bond network contains closed rings, with substantial populations of five- and six-membered H-bond rings [11]. What remains less clear is whether the reported time-domain and frequency-domain features admit a common discrete ordering.

Ultrafast and picosecond hydrogen-bond dynamics provide a reported set of characteristic timescales and intermolecular band peaks, from intramolecular O–H stretch (∼10 fs) and H–O–H bend (∼1640 cm^−1^ in liquid water, period ∼ 20 fs [12]) through H-bond stretch and bend bands at 60–180 cm^−1^ (periods ∼ 185–560 fs), picosecond rearrangements, and tens-of-picoseconds orientational decorrelation [13,14,15,16,17]. We use these literature values as reported band centres and characteristic timescales, not as unique microscopic mode assignments. In parallel, water shows a cooperative thermodynamic anomaly with the density maximum at 277.15 K and a first-order freezing transition at 273.15 K.

Existing descriptions of liquid-water dynamics usually address one observable class at a time: INM studies analyse vibrational eigenvectors and mode localisation [18,19,20], empirical and machine-learned potentials provide simulation Hamiltonians [21,22,23], phonon-like analyses describe propagating collective modes [24], and two-state or two-liquid models target thermodynamic anomalies [25,26,27]. The present work asks a different, more limited question: whether the time-domain H-bond processes [13,15,16,17] and the far-IR/THz bands [9,10,28] can be placed on one externally fixed discrete ladder, and whether the corresponding participation content can be measured directly in MD.

For the near-freezing anomaly specifically, percolation models [29,30], locally favoured structures [26,27], and two-liquid pictures [25,31,32,33] provide the relevant thermodynamic context. The temperature map below is therefore presented as a calibrated ladder relation, not as a replacement equation of state.

The model developed below uses these literatures in a specific division of roles: ultrafast and THz experiments supply the timescale and band inputs [13,14,15,16,17,34,35], graph theory supplies the loop-free shell count tested against the H-bond graph [11,36], and INM analysis supplies the participation measurement used to populate the ladder dynamically [18,19,20,24].

The theoretical scaffold of the approach is summarised here in physical terms before it is developed formally. The defining equations are deferred to Section 2, and this overview states only the ideas and their logical order, so that the physical picture and the formalism need not be absorbed at once. The scaffold rests on three ingredients and one chain that links them. *(1) A loop-free reference graph.* Because each water molecule donates and accepts two hydrogen bonds, its local hydrogen-bond coordination is four, and the maximally branching graph consistent with that valence that contains no closed rings is the tetrahedral Bethe lattice. In such a graph, the number of molecules reachable within a given number of hydrogen-bond steps of a central molecule grows geometrically with that number of steps, precisely because rings, which would fold distant branches back onto one another and slow the growth, are excluded by construction. This loop-free graph is therefore a reference bound rather than a structural model of real water, whose network contains many such rings, and its precise role is developed in Section 2. *(2) A discrete geometric ladder.* The characteristic timescales are compared against a geometric sequence of reference periods whose successive rungs differ by a fixed multiplicative factor, the golden ratio φ. This ratio is supplied externally by a separate theoretical framework [37,38] and is not fitted to water. Only a single timescale is taken from experiment to set the absolute position of the ladder. *(3) A physical bridge between the two.* A shell of co-moving molecules is treated as one collective harmonic oscillator whose effective mass grows in proportion to the number of molecules that actually participate, so that heavier, more collective motions correspond to longer periods. The logical chain therefore runs from *topology* (how many molecules a shell could engage) to *participation* (how many actually share the motion) to *timescale* (its period, placed on the ladder). The decisive, model-independent step is the middle one: whether the participation really keeps growing with shell size, as the small-cluster picture assumes, or instead saturates, is not postulated but measured directly in the molecular-dynamics analysis of Section 5, and it is that measurement, not the ladder, that carries the physical conclusion of the paper.

This paper pursues three linked aims. Aim 1 places the reported ultrafast H-bond timescales and the principal far-IR/THz bands on the rung windows of the ladder $\tau_{n}=\tau_{0}\varphi^{n}$, indexed by the loop-free Bethe-shell sizes N(n). Aim 2 uses the same ladder, with one time anchor and one empirical energy anchor, to construct a calibrated map of the freezing and density-maximum temperatures. Aim 3 tests the dynamical content of the shell assignment by measuring, in liquid water, how many molecules share each low-frequency vibration. These aims deliberately separate organization from prediction: the bracketing is not claimed to prove uniqueness of φ, and the thermodynamic map is treated as calibrated.

The physical question underlying this study is mechanistic rather than numerical. The low-frequency dynamics of liquid water (the intermolecular vibrations in the far-infrared/terahertz region and the picosecond hydrogen-bond rearrangements) are collective motions of hydrogen-bonded molecules, not motions of isolated molecules. Their character is therefore controlled by how far mechanical coherence extends through the hydrogen-bond network: whether a given low-frequency vibration is a compact breathing motion of a molecule and its nearest neighbours, or a delocalised motion shared across many molecules. The central physical result of this paper is a direct measurement of that coherence range. An instantaneous-normal-mode analysis of TIP4P/Ew water shows that the low-frequency intermolecular modes are delocalised, and that the number of molecules sharing a mode saturates at about one coordination shell instead of growing with the size of the region formally assigned to each band. This picture agrees with independent experimental evidence: two-dimensional terahertz–infrared–visible spectroscopy, together with the accompanying simulations, identifies the ∼60 cm^−1^ hydrogen-bond-bending and ∼180 cm^−1^ hydrogen-bond-stretching bands of liquid water as collective, delocalised motions of the hydrogen-bond network [39]. This conclusion rests on the measured vibrational eigenvectors alone and does not depend on the golden-ratio ladder or on the loop-free idealisation used to order the timescales.

Within that physical setting, the golden-ratio ladder and the loop-free Bethe sequence play a deliberately limited, auxiliary role, and the present work is not a mathematical construction in search of a physical system. The Bethe lattice is not proposed as a structural model of liquid water. It is used only as the loop-free reference graph of tetrahedral local valence (the maximally branching connectivity consistent with two donor and two acceptor directions per molecule), and it serves two purposes: it provides an analytic upper bound on how quickly a coherent region can grow with hydrogen-bond distance, and it supplies an integer shell index against which the observed timescales and band centres can be ordered. The real network departs from this reference through ring closure and disorder, and measuring that departure is one purpose of the molecular-dynamics tests below. The ladder likewise contributes only the rung spacing used to order the data. The physical content that the data carry, namely the delocalisation-saturated participation law, is measured independently of both the ladder and the tree.

The remaining part of the paper is organized to follow these three aims in turn. Section 2 defines the algebraic inputs, the physical bridges, and the two calibration anchors. In Section 3, we develop Aim 1, including a quantitative statistical assessment of the ladder (Section 3.2) that scans alternative logarithmic bases, applies a null-model test and a spectral (Rayleigh) test for a preferred spacing, and reports the sensitivity to the timescale uncertainties and to the time anchor. Section 4 develops Aim 2, and Section 5 develops Aim 3. In Section 6, we discuss open questions and the outlook. We conclude in Section 7.

## 2. Model Construction

### 2.1. Input Parameters

Table 1 collects the recurring symbols used in the model and in the MD tests.

**Table 1:** Nomenclature for the main quantities used in the model and tests.

Symbol	Meaning	Where It Enters
φ	Golden-ratio spacing, $(1+\sqrt{5})/2$	Ladder definition, Equation (1)
*n*	Integer rung or Bethe-shell index	Rung windows and shell depth
$\tau_{0}$	Base timescale fixed by the 80 fs anchor, $\tau_{0}=80\;\mathrm{fs}\cdot \varphi^{-5}\approx 7.2$ fs	Equation (1)
$\tau_{n}$	Period assigned to rung *n*	$\tau_{n}=\tau_{0}\varphi^{n}$
*z*	Ideal local H-bond valence	z=4
N(n)	Loop-free cumulative Bethe-shell size	Equation (2) and Table 2
$N_{\mathrm{eff}}$	Effective participation number	Equation (4)
$P_{k}$	INM participation ratio of mode *k*	Dynamical test, Equation (5)
$N_{\mathrm{MD}}\left(n\right)$	Measured cumulative BFS neighbour reach in the H-bond graph	Topological test, Section 5.2
$E_{\mathrm{coh}}$	Empirical energy anchor, $\varphi^{-5}$ eV	Section 4
$T_{c}$	Cooperative-transition temperature bracket	Equation (14)
$T_{\max}$	Density-maximum temperature bracket	Equation (15)

**Table 2:** z=4 Bethe-lattice shells with predicted rung ranges and experimental matches across both ultrafast (time-domain) and THz/far-IR (frequency-domain) sets. Entries in the “Pred. rung”, “Pred. $\tilde{\nu }$”, and “Pred. τ” columns are derived from Equation (2) (Bethe-shell size N(n)), Equation (13) (the $\tau \propto \sqrt{N_{\mathrm{eff}}}$ scaling), and the rung-5 calibration anchor (80 fs). The “Experimental match” column lists independent literature measurements [7,8,9,10,13,14,15,17]. The φ-bracket column uses open intervals $\left(\varphi^{a},\varphi^{b}\right)$. This table reports calibration-free placements on the ladder.

Shell	N(n)	φ Bracket	Pred. Rung	Pred. $\tilde{\nu }$ (cm^−1^)	Pred. τ	Experimental Match (cm^−1^, τ)
0	1	$\varphi^{0}$	5	417	80 fs	calibration anchor: rung 5 ≡ 80 fs ≡ 417 cm^−1^
1	5	$\left(\varphi^{3},\varphi^{4}\right)$	6–7	160–260	129–209 fs	THz H-bond stretch: ∼180 cm^−1^, ∼185 fs
2	17	$\left(\varphi^{5},\varphi^{6}\right)$	7–8	100–160	209–339 fs	ultrafast vibr. relaxation: 111 cm^−1^, 300 fs
3	53	$\left(\varphi^{8},\varphi^{9}\right)$	9–10	38–61	549–887 fs	THz H-bond bend: ∼60 cm^−1^, ∼555 fs
4	161	$\left(\varphi^{10},\varphi^{11}\right)$	10–11	24–40	0.89–1.44 ps	ultrafast H-bond breaking: 33 cm^−1^, 1 ps
5	485	$\left(\varphi^{12},\varphi^{13}\right)$	11–12	15–24	1.44–2.33 ps	THz low-freq. connectivity: ≲30 cm^−1^, ≳1.1 ps
6	1457	$\left(\varphi^{15},\varphi^{16}\right)$	12–13	9–15	2.33–3.76 ps	ultrafast molecular jump: 13 cm^−1^, 2.5 ps
7	4373	$\left(\varphi^{17},\varphi^{18}\right)$	13–14	6–9	3.76–6.09 ps	ultrafast freq. decorrelation: 6.7 cm^−1^, 5 ps

Our model invokes two algebraic parameters. The first is the ladder spacing golden ratio parameter $\varphi =(1+\sqrt{5})/2$, The value φ is an external input: it is fixed by the separate theoretical framework of Refs. [37,38,40,41,42]. Everything below tests how well water’s timescales are organized by this externally fixed spacing. The ladder spacing is therefore fixed before any comparison with the water data, and the analysis that follows tests an externally specified hypothesis rather than a spacing fitted to the observations. The value φ was not adjusted to improve the agreement with water. Because φ is supplied externally, the relevant question is not whether water selects it but whether the externally fixed value is consistent with the observed timescales. Section 3 quantifies how the placement shifts under the uncertainty of the time anchor and shows that φ sits comfortably within the range of spacings compatible with the data. The fit quality is a shallow, structureless function of the base, so with only a handful of log-spaced timescales, the bracketing has limited power to distinguish φ from nearby log-period spacings. This reflects the resolving power of a small data set rather than evidence against φ, and it makes explicit how much of the ordering is specific to φ and how much would follow from any comparable log-period spacing. It defines the discrete timescale ladder

(1) $$\tau_{n}=\tau_{0}\;\varphi^{n}$$

where $\tau_{n}$ is the characteristic timescale (the logarithmic “period”) assigned to rung *n* of the ladder, and $\tau_{0}$ is the unit/base timescale (the rung-0 period), which sets the absolute scale.

**Figure 1:**
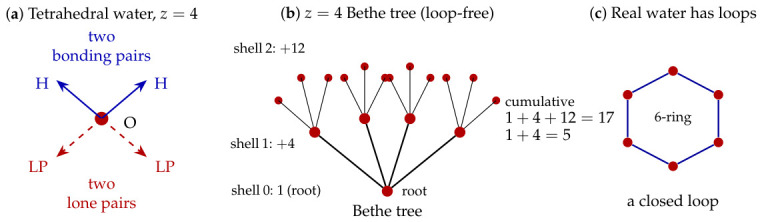
Schematic of the model’s structural input. (**a**) Tetrahedral water with two donor bonds and two lone-pair acceptor directions. (**b**) The corresponding loop-free z=4 Bethe-tree idealisation. Layer counts 1+4+12=17 are the cumulative shell sizes that enter Equation (2) at z=4 (this is N(2)=17 in the sequence of Equation (3)). (**c**) A 6-membered H-bond ring, illustrating the loop structure absent from the Bethe lattice.

The second input parameter is the cumulative Bethe-lattice shell size sequence,

(2) $$N\left(n\right)=1+z\sum_{k=0}^{n-1}{\left(z-1\right)}^{k}=2\cdot 3^{n}-1$$

evaluated at the ideal tetrahedral valence z=4 [5,6]. It is the local valence of the loop-free reference graph, used because an isolated water molecule has two donor and two acceptor H-bond directions. The tetrahedral local geometry is illustrated in Figure 1a and feeds directly into the branching number z−1=3 of the loop-free Bethe reference. In Equation (2), N(n) is the total number of vertices reachable from a chosen root within *n* H-bond steps, including the root itself and all preceding shells. Equation (2) is the cumulative shell count for a rooted neighbourhood of the infinite *z*-regular Bethe lattice. A finite rooted tree with the same branching is often called a Cayley tree. Here we use only the local, loop-free shell counts, not boundary properties of a finite tree. For n=0,…,7 this yields the sequence

(3) N(n)∈{1,5,17,53,161,485,1457,4373}.

### 2.2. Participation Number $N_{eff}$

We introduce the *effective participation number* $N_{\mathrm{eff}}$ as a standard inverse-participation-ratio (IPR) measure of how many molecules carry significant amplitude in a vibrational mode. For a normalised eigenvector with components $u_{i}$ on molecule *i* ($\sum_{i}u_{i}^{2}=1$),

(4) $$N_{\mathrm{eff}}\;\equiv \;\frac{{\left(\sum_{i}u_{i}^{2}\right)}^{2}}{\sum_{i}u_{i}^{4}}\;=\;\frac{1}{\sum_{i}u_{i}^{4}}$$

This equation gives $N_{\mathrm{eff}}=1$ for a mode localised on one molecule, $N_{\mathrm{eff}}=N_{\mathrm{tot}}$ for a mode spread uniformly over the whole box, and $N_{\mathrm{eff}}=k$ for an ideal mode equally shared by *k* molecules. The INM test of Section 5.3 evaluates this same quantity on mass-weighted Hessian eigenvectors $\left\{v_{k}\right\}$ as

(5) $$P_{k}\;=\;\frac{1}{\sum_{i}v_{ki}^{4}}$$

so $N_{\mathrm{eff}}$ and $P_{k}$ are the same participation measure, used respectively for the model assignment and the simulated eigenvectors. We assume that the shell-*n* participation number equals the Bethe-shell size,

(6) $$N_{\mathrm{eff}}\left(n\right)\;=\;N\left(n\right)\;=\;2\cdot 3^{n}-1$$

and call the corresponding idealised oscillation the *shell-n vibration*: a chosen central molecule and its first *n* H-bond shells are assumed to move in phase. For shells beyond the first neighbour cage, this is a formal model construct, not a claim that hundreds of molecules in the disordered liquid execute a compact in-phase breathing motion. The MD test below is included precisely to determine where that compact-coherence picture breaks down. This assumption is tested directly against the molecular-dynamics participation data in Section 5.3.

Real liquid water differs from the loop-free Bethe idealisation in two ways: it has loops (the five- and six-membered rings noted in the Introduction) and finite correlation lengths. The Bethe lattice is therefore retained only as the loop-free topological reference for local tetrahedral branching, not as a literal structural model. The reason for adopting a loop-free reference, rather than a more realistic ringed network, is physical. For a purely branching connectivity the number of molecules within *n* hydrogen-bond steps grows as fast as the local valence permits, so N(n) is an upper bound on the number of molecules that a shell-*n* breathing motion could engage. Ring closure and coordination deficits in the real liquid can only reduce that number. The loop-free sequence thus isolates a single, well-defined limiting case (maximal coherent growth) whose breakdown in the real network is precisely the quantity measured in Section 5. Fixing the reference at the ideal tetrahedral valence z=4 keeps it tied to the molecular geometry of water rather than to an adjustable coordination number, so that any disagreement with the simulation is attributable to network structure and not to a tuned input. Loop-free and loop-containing tree substrates (Bethe and Husimi lattices) have been used in the same reference spirit to model the hydrogen-bond network of liquid water, reproducing its coordination-defect statistics and thermodynamic anomalies [43]. The present use of the loop-free Bethe sequence is the minimal member of that family, and the loop-containing (Husimi) extension is the natural next step identified in Section 6. Figure 1 illustrates the model: panel (a) shows the molecular geometry, panel (b) the resulting Bethe tree with layer counts 1+4+12=17, and panel (c) a six-membered ring that is forbidden in the Bethe lattice but present in real water.

### 2.3. Calibration Anchors

Two empirical anchors fix the absolute scales used in this paper: a time anchor for the spectroscopic ladder and an energy anchor for the temperature map.

First, the reported ∼80 fs H-bond contraction time of liquid water [13,15,17] sets the unit timescale

(7) $$\tau_{0}\;=\;80\;\mathrm{fs}\;\cdot \;\varphi^{-5}$$

Anchoring rung 5 (rather than another integer rung) is a labelling convention only: the ladder $\tau_{n}=\tau_{0}\;\varphi^{n}$ is invariant under the replacement of *n* by n+k together with $\tau_{0}$ by $\tau_{0}\;\varphi^{-k}$, so only the inter-rung gaps between successive assigned timescales are physical. Appendix B checks the shell-1 H-bond stretching assignment in TIP4P/Ew water. Because the sub-100 fs water response is obtained from multi-exponential spectroscopic fits and can mix spectral diffusion, librational, and structural components, the 80 fs value is used here as a literature anchor with uncertainty rather than as a sharp microscopic constant. The operational consequences of the anchor (rung-offset formula, rung-assignment criterion) are developed in Section 2.4.

Second, as an empirical energy anchor we set

(8) $$E_{\mathrm{coh}}\;\equiv \;\varphi^{-5}\;\mathrm{eV}\;\approx \;0.0902\;\mathrm{eV}$$

Assigning the unit electronvolt to the dimensionless $\varphi^{-5}$ is a chosen convention. we adopt it because the resulting value (≈0.09 eV) sits near the low end of the measured per-bond H-bond enthalpy range [0.08,0.30] eV [44,45,46,47], and we quantify below how the temperature mapping depends on this choice. The dependence of the freezing-point bracket on this choice is quantified in Section 4.

### 2.4. Vibrational Timescale τ

This subsection discusses a connection between the participation number $N_{\mathrm{eff}}$ and the vibrational timescale τ, and then states how individual timescales and whole Bethe shells are assigned to integer rungs of the φ-ladder. It proceeds in four steps: Section 2.4.1 states the two physical assumptions, Section 2.4.2 derives the period law $\tau \propto \sqrt{N_{\mathrm{eff}}}$ from them, Section 2.4.3 develops the consequence of combining that law with the bridge $N_{\mathrm{eff}}\left(n\right)=N\left(n\right)$, and Section 2.4.4 gives the rung-assignment criterion and the Bethe-shell windows it produces. The assumptions are therefore part of the ideal ladder construction. They are not assumed to survive unchanged in the real liquid. Section 5.3 measures the actual participation and shows that the equal-amplitude compact-cluster reading is replaced by a saturated, delocalised one.

#### 2.4.1. Assumptions

The shell-*n* vibration is modelled as a coherent harmonic oscillation under two assumptions. (i) All $N_{\mathrm{eff}}$ molecules share a single collective coordinate X(t) and oscillate with equal displacement amplitude ($u_{i}=1/\sqrt{N_{\mathrm{eff}}}$ for all *i*, and hence equal velocity amplitude $\dot{X}$). (ii) The restoring stiffness of that coordinate is a shell-independent effective H-bond stiffness *k*, with no $N_{\mathrm{eff}}$-dependent renormalisation. Assumption (i) is more restrictive than the general IPR definition of Section 2.2: for non-uniform amplitudes, the effective mass is smaller than $N_{\mathrm{eff}}\;m$ (the most active molecules dominate), so $\tau \propto \sqrt{N_{\mathrm{eff}}}$ is then only a period *upper bound*. Under the equal-amplitude assumption (i), the inequality becomes an equality and $\tau \propto \sqrt{N_{\mathrm{eff}}}$ is exact within the model.

#### 2.4.2. Period Scaling $\tau \propto \sqrt{N_{\mathrm{eff}}}$

Under assumption (i), the total kinetic energy is

(9) $$\mathrm{KE}=\frac{1}{2}\sum_{i=1}^{N_{\mathrm{eff}}}m\;{\dot{x}}_{i}^{\;2}=\frac{1}{2}\;\left(N_{\mathrm{eff}}\;m\right)\;{\dot{X}}^{2}$$

so the effective mass of the collective coordinate is the sum of the individual molecular masses,

(10) $$M_{\mathrm{eff}}=N_{\mathrm{eff}}\;m\;\propto \;N_{\mathrm{eff}}$$

where $m=m_{H_{2}O}=18.015$ amu. With the shell-independent stiffness *k* of assumption (ii), Newton’s equation for *X* reads

(11) $$M_{\mathrm{eff}}\;\ddot{X}=-k\;X$$

giving an angular frequency $\omega =\sqrt{k/M_{\mathrm{eff}}}$ and a period

(12) $$\tau \;=\;\frac{2\pi }{\omega }\;=\;2\pi \sqrt{\frac{N_{\mathrm{eff}}\;m}{k}}\;=\;\sqrt{N_{\mathrm{eff}}}\;\cdot \;\tau_{\mathrm{single}},\;\tau_{\mathrm{single}}\equiv 2\pi \sqrt{m/k}$$

We take this as the working scaling of the model:

(13) $$\tau \propto \sqrt{N_{\mathrm{eff}}}$$

#### 2.4.3. Shell-Vibration Assignment and the Per-Rung Gap

Combining the period law of Section 2.4.2 with the participation-bridge identification Equation (6) gives $\tau_{n}\propto \sqrt{N(n)}$, whose shell-to-shell growth is close to, but not exactly, the φ-ladder spacing. The size of this near-coincidence is definite. Because the shell sizes grow as $N\left(n\right)=2\cdot 3^{n}-1∼3^{n}$, successive shells differ by the branching factor z−1=3, so the period law $\tau \propto \sqrt{N(n)}$ gives an asymptotic period ratio $\tau_{n+1}/\tau_{n}=\sqrt{N(n+1)/N(n)}$ that approaches $\sqrt{3}\approx 1.732$ per shell, while the ladder spaces by φ≈1.618 per rung. The residual per-step factor $\sqrt{3}/\varphi \approx 1.071$ accumulates to ${\left(\sqrt{3}/\varphi \right)}^{8}\approx 1.73$ over eight shells and to $\sqrt{N(8)}/\varphi^{8}\approx 2.4$ across the reported set. A single-rung-wide (point) ladder would require a reference sequence whose growth rate is exactly $\varphi^{2}$ per shell, so that $\sqrt{N(n+1)/N(n)}$ approaches φ. The z=4 Bethe sequence does not have this growth rate, and we keep z=4 because it is fixed by the tetrahedral chemistry. The consequence is that each Bethe shell is bracketed by a two-rung-wide window rather than a single rung, and only shell 0 lands on one rung, since $N\left(0\right)=1=\varphi^{0}$. Each shell should therefore be read as falling within a two-rung-wide band of the ladder, not as hitting a single sharp $\varphi^{n}$ value.

#### 2.4.4. Rung-Assignment Criterion and Bethe-Shell Windows

The ladder is anchored at $\tau_{0}=80\;\mathrm{fs}\cdot \varphi^{-5}\approx 7.2$ fs. We state here the rung-assignment criterion that compares individual timescales and Bethe-shell sizes to the ladder.

The rung offset of a mode with participation number $N_{\mathrm{eff}}$ relative to rung 5 follows from Equation (13) and the calibration $\tau \left(N_{\mathrm{eff}}=1\right)=\tau_{0}\varphi^{5}$: since $\tau \propto \sqrt{N_{\mathrm{eff}}}$ and $\tau \propto \varphi^{n}$, the rung shift satisfies $\varphi^{\Delta r}=\sqrt{N_{\mathrm{eff}}}$, giving $\Delta r=\frac{1}{2}\log_{\varphi }N_{\mathrm{eff}}$.

We adopt the following formal rung-assignment criterion: a measured timescale $\tau_{\mathrm{obs}}$ is said to be *assigned to rung n* if $\tau_{\mathrm{obs}}\in \left[\tau_{0}\;\varphi^{n-1/2},\;\tau_{0}\;\varphi^{n+1/2}\right)$, i.e., if its log-period satisfies $|\log_{\varphi }\left(\tau_{\mathrm{obs}}/\tau_{0}\right)-n|<1/2$. This gives each rung a multiplicative window of width φ≈1.62 in period, with a half-rung tolerance of $\sqrt{\varphi }\approx 1.27$ (about ±27%) around the centre. The tolerance is wide, so the bracketing claim is qualitative. This nearest-integer rule defines what we mean by “a rung match”. Note that the Bethe-shell windows span two rungs because each N(n) lies between two consecutive φ powers. The half-rung criterion applies to individual observed timescales, not to the Bethe-shell brackets.

Each Bethe-shell size N(n) is then bracketed by consecutive φ powers, $\varphi^{a}<N\left(n\right)<\varphi^{b}$, and Equation (13) places the predicted integer rung in the window [5+⌊a/2⌋,5+⌈b/2⌉]. Concretely, $\varphi^{3}<5<\varphi^{4}$ (shell 1 gives rungs 6–7), $\varphi^{8}<53<\varphi^{9}$ (shell 3 gives rungs 9–10), and $\varphi^{12}<485<\varphi^{13}$ (shell 5 gives rungs 11–12). These are the three THz-matching windows tabulated and discussed in Section 3.1. Section 2.4.1, Section 2.4.2, Section 2.4.3 and Section 2.4.4 thus turn the algebraic inputs of Section 2 into a candidate timescale ladder for liquid water.

## 3. Rung Bracketing of the H-Bond Hierarchy

**Figure 2:**
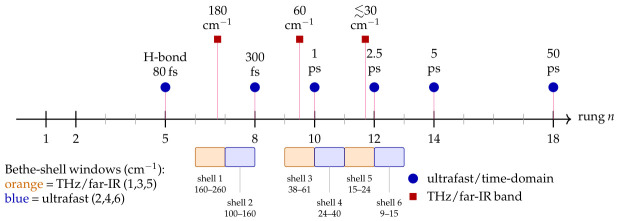
Rung-ladder map of the water timescale set. Integer rungs are logarithmic periods $\tau_{n}=\tau_{0}\varphi^{n}$ relative to the 80 fs calibration at rung 5. Circles mark time-domain Bethe-shell assignments, squares mark THz/far-IR Bethe-shell bands, orange windows show the THz/far-IR Bethe-shell ranges (odd shells 1, 3, 5), and blue windows show the ultrafast Bethe-shell ranges (even shells 2, 4, 6). The intramolecular O–H stretch and H–O–H bend are intramolecular vibrations, not shell-*n* network modes of the Bethe construction, and are excluded from this figure and from all ladder-performance claims (see Appendix A). The panel is read as an ordering diagram: the horizontal position of each marker is its logarithmic period relative to the 80 fs anchor at rung 5, and the shaded bands are the two-rung windows predicted for each Bethe shell. The vertical axis carries no physical meaning and only separates the time-domain (circles) and frequency-domain (squares) sets for legibility.

This section brackets the reported H-bond timescale hierarchy and the principal far-infrared/THz band centres against the discrete logarithmic ladder $\tau_{n}=\tau_{0}\;\varphi^{n}$ of Section 2. Periods and wavenumbers are related by $\tau =1/(\tilde{\nu }c)$, equivalently $\tau \tilde{\nu }=1/c\approx 33,356\;\left[\mathrm{fs}\right]/\left[\mathrm{cm}\right]$. The master rung table is Table 2. The graphical summary is Figure 2.

### 3.1. Master Rung Table and Consistency Checks Against the Data

Applying the rung-bracketing rule of Section 2.4 to the eight Bethe-shell sizes $N\left(n\right)=2\cdot 3^{n}-1$ (n=0,…,7) yields the master rung table (Table 2). Each row gives one shell, its φ bracket, the predicted integer rung window, the predicted period and wavenumber range, and the independent literature measurement that falls in that window. The rung-5 row (shell 0, $N_{\mathrm{eff}}=1$) is the calibration anchor $\tau_{0}$ and is fixed by construction. The remaining rows are calibration-free placements on the same ladder. They show how the reported H-bond timescales and band centres align with the Bethe-indexed rung windows, while their statistical status is discussed after Figure 2.

Because the construction of Table 2 is central to the paper, we set out explicitly how each column is obtained; every entry follows from the two Bethe/ladder inputs and a single experimental anchor, with no per-row fitting. Column 1 lists the shell index *n*, and column 2 (“N(n)”) its loop-free Bethe size $N\left(n\right)=2\cdot 3^{\;n}-1$ from Equation (2), i.e., 1,5,17,53,161,… for n=0,1,2,3,4,…. The “φ bracket” column is obtained purely arithmetically: each integer N(n) is enclosed between the two consecutive integer powers of the golden ratio that straddle it, $\varphi^{a}<N\left(n\right)<\varphi^{b}$ with b=a+1. For example, $\varphi^{3}\approx 4.24<5<6.85\approx \varphi^{4}$ gives the bracket $\left(\varphi^{3},\varphi^{4}\right)$ for shell 1, and $\varphi^{8}\approx 47.0<53<76.0\approx \varphi^{9}$ gives $\left(\varphi^{8},\varphi^{9}\right)$ for shell 3. The “Pred. rung” window then follows from the period law $\tau \propto \sqrt{N_{\mathrm{eff}}}$ of Equation (13), which maps a shell of size N(n) to the rung interval [5+⌊a/2⌋,5+⌈b/2⌉] measured from the rung-5 anchor (Section 2.4); the factor $\frac{1}{2}$ is the square root in the period law, and the two-rung width is the direct consequence of N(n) falling between two φ powers rather than on one. The “Pred. $\tilde{\nu }$” and “Pred. τ” columns are the same window expressed as a wavenumber and a period through $\tau_{n}=\tau_{0}\varphi^{\;n}$ and $\tau =1/(\tilde{\nu }c)$, using the single anchor $\tau_{0}$ (rung 5 ≡80 fs). Only the last column, “Experimental match”, comes from outside the construction: it lists the independently reported ultrafast timescale or far-infrared/THz band centre [7,8,9,10,13,14,15,17] that falls in the predicted window, and no experimental value is used to place any row except the rung-5 anchor.

The entries in Table 2 fall into three classes:

(i) THz/far-IR bands: The three principal intermolecular bands of liquid water are associated with the three odd-numbered Bethe shells 1, 3, and 5, namely the H-bond stretch, H-bond bend, and low-frequency connectivity band [7,8,9,10].
(ii) Ultrafast and picosecond timescales: Shells 2, 4, 6, and 7 bracket the reported H-bond relaxation processes from hundreds of femtoseconds to several picoseconds [13,14,15,17].
(iii) One outside-shell rung: Rung 18 carries the network orientational decorrelation (50 ps), the slowest reported H-bond timescale. Numerical placements for the intramolecular O–H stretch and H–O–H bend are recorded separately in Appendix A.

The eight Bethe shells span rungs 5–14 of the ladder (about six octaves of timescale). The graphical summary on a logarithmic rung axis is Figure 2.

The rung axis of Figure 2 is not uniformly filled: a bare rung separates the shell-2 and shell-3 windows. This follows directly from the period law $\tau \propto \sqrt{N_{\mathrm{eff}}}$, which places a shell at rung $5+\frac{1}{2}\log_{\varphi }N\left(n\right)$. A shell would fall on rungs 8–9 only if its size lays in the interval $\varphi^{6}\approx 18<N<\varphi^{8}\approx 47$, but the Bethe shells grow by a factor of three per step, so shell 2 has N=17 (just below $\varphi^{6}$, rungs 7–8) and shell 3 has N=53 (just above $\varphi^{8}$, rungs 9–10). The size jumps straight from 17 to 53, stepping over the whole [18,47] range that brackets $\varphi^{7}\approx 29$, so no shell lands near rung 8.5 and that rung stays empty. The gap is the per-rung mismatch of Section 2.4.3 seen graphically: each shell advances the window centre by only $\frac{1}{2}\log_{\varphi }3\approx 1.14$ rungs, but because N(n) grows in discrete ×3 jumps, the bracket exponents step through 5,6,8,9,… and occasionally skip a φ-power. The shell-3 to shell-6 windows happen to abut without skipping an integer rung, so the gap appears only between shells 2 and 3.

Using the half-rung criterion of Section 2.4.4, the model brackets the timescale set but does not predict exact $\varphi^{n}$ ratios. Excluding the shell-5 connectivity entry, which the literature reports only as a bound (≲30 cm^−1^, ≳1.1 ps), the seven well-defined non-anchor assignments have mean |deviation|≈12% and maximum ≈20% (rung 18, 50 ps). A full statistical assessment using all nine intermolecular timescales is given in Section 3.2. Table 2 and Figure 2 therefore establish a bounded placement result: the reported timescales and band centres fall inside their assigned rung windows from one time anchor. Given the window widths, the anchor uncertainty, and the retrospective band assignments, this bracketing is best read as a consistent organizing scheme rather than, on its own, a model-selection result that singles out one logarithmic base over its neighbours.

*Sensitivity to the anchor.* The 80 fs contraction time is itself a model-dependent quantity, extracted from multi-exponential fits to 2D-IR spectral-diffusion and photon-echo data and conflating several sub-100 fs processes, with a realistic spread of order ±20 fs (±25%) [13,17]. Because of the relabelling invariance noted in Section 2.3, changing the anchor leaves the inter-rung gaps unchanged but shifts all absolute rung labels. A ±25% shift in $\tau_{0}$ corresponds to $\Delta r=\log_{\varphi }\left(1.25\right)\approx 0.46$ rungs, about half a rung, so assignments near a window edge can move by one full integer label under the stated anchor uncertainty. Consequently the integer labels in Table 2 should be read as central assignments. The robust statement is the relative ordering of the reported processes across the ladder, whereas any individual edge-case label can shift by roughly one rung under the anchor envelope.

The probability that these placements arise by chance requires an explicit statement, because the alignment of a small set of timescales with ladder rungs could otherwise be over-interpreted as a numerical coincidence. Two facts bound that probability. First, the half-rung windows tile the logarithmic period axis without gaps, so every timescale in the observed range falls inside some rung window with probability one. The statement that the reported timescales “fall on rungs” therefore describes the ordering rather than serving as independent evidence for the construction. Second, for the three far-infrared/terahertz bands, which fall into their assigned two-rung windows, a uniform-in-log null model over the observed four-octave range gives a per-band probability of about 0.34 and a nominal joint probability of order 0.03–0.05 for three independent bands. Once the retrospective freedom to assign three bands among the eight available shells is accounted for (of order 83=56 choices), this nominal significance is inflated well above the 0.05 level. With only a handful of log-spaced timescales, the bracketing is therefore best read as a consistent organising scheme rather than as a statistically decisive result. The load-bearing, falsifiable physical result of the paper is the measured participation law of Section 5, which is obtained directly from the vibrational eigenvectors and is not a coincidence of numbers. Two further considerations frame this caution. Discrete scale invariance, and the log-periodic ordering it implies, is a recognised feature of hierarchical physical systems [48], so a golden-ratio (log-periodic) ladder is a physically meaningful organising hypothesis rather than mere numerology. Its presence in data must nonetheless be demonstrated rather than assumed. At the same time, it is well documented that logarithmic sampling combined with cumulative or multi-exponential fitting can generate *artifactual* log-periodic structure even in data without any underlying hierarchy [49]. Both considerations are why the present work presents the bracketing as a consistent organising scheme, appropriate for a data set of this size, and rests its physical conclusions on the direct participation measurement.

### 3.2. Quantitative Statistical Assessment of the Ladder

The preceding paragraphs state the statistical status of the bracketing in words. This subsection makes it quantitative, so that the strength of the “golden-ratio” language can be assessed directly rather than taken on trust. Five checks are reported, all of them applied to the same fixed set of intermolecular observables and none of them requiring new simulations: a comparison against alternative logarithmic bases, a null-model randomisation, an interval test using the timescale uncertainties, an anchor-sensitivity analysis, and a spectral (Rayleigh) test for a statistically preferred spacing.

*Data set and selection rule.* The checks use the nine intermolecular timescales of Table 2, obtained under one explicit rule: one representative period per distinct H-bond process or far-infrared/terahertz band, taken from the cited ultrafast and THz literature [7,8,9,10,13,14,15,17], with the two intramolecular vibrations (O–H stretch, H–O–H bend) excluded because they are not shell-*n* network modes (Appendix A). Each value carries a plausible fractional range of ±20–30%, reflecting the spread of reported central values across those sources rather than a single formal error bar. These ranges are used in the interval test below.

*(i) Comparison with alternative logarithmic bases.* We replaced φ by a general base *b*, re-anchored the ladder in each case through the single continuous offset that minimises the residual, and recomputed the fit quality as both the root-mean-square residual to the nearest rung (in rung units, where 0 is a perfect snap and 0.5 is the worst case) and the mean and maximum fractional period deviation. Table 3 reports the result. Across the entire range b∈[1.40,1.90], the mean absolute period deviation stays between about 6% and 13% and the rung residual between about 0.17 and 0.29: the fit quality is a shallow, structureless function of the base, and the variations across the whole interval are small and, as the spectral test of check (v) below shows, not statistically significant. It is essential to the logic of the paper that the base is not a free parameter tuned to water. It is fixed externally by the separate theoretical framework [37,38], so the relevant question is not which base minimizes the residual, but whether the externally supplied φ is consistent with the observed timescales. It is: φ lies well inside the broad plateau of acceptable bases, and the small metric differences between φ and neighboring values carry no statistical weight (check (v) below). The data therefore single out neither φ nor any competing base, and we make no claim that liquid water “selects” the golden ratio.

The practical consequence of replacing φ by an adjacent number is therefore small and fully contained in Table 3. Moving the base from φ≈1.618 to a neighbouring value such as 1.5, 1.7, or $\sqrt{3}\approx 1.732$ changes the error metrics only marginally and never degrades the bracketing qualitatively, because the whole interval b∈[1.40,1.90] lies on the same shallow plateau and, as check (v) shows, the differences between bases across this interval are not statistically significant. An adjacent spacing thus reorganises the integer rung labels but leaves every physical conclusion of the paper unchanged, since those conclusions rest on the measured participation law and not on the numerical value of the ladder base.

*(ii) Null-model test.* To test whether the observed bracketing is unusual, we generated $2\times 10^{5}$ synthetic data sets, each of nine timescales drawn uniformly in log-period over the observed range (80 fs to 50 ps), and fitted every draw to the φ-ladder with the same optimal-offset procedure. The water set has RMS rung residual 0.27. The null distribution has median residual 0.23, and about 93% of random draws are bracketed at least as tightly as the real water timescales. The corresponding chance probability, p≈0.93, reflects the limited resolving power of a nine-timescale set: with so few log-spaced timescales, a test of this kind does not, on its own, set the ladder apart from a generic log-spaced spectrum, which is a property of the sample size rather than a shortcoming of the ladder. This is consistent with the window-tiling argument given above and with the retrospective assignment penalty affecting the three THz bands.

*(iii) Sensitivity to the timescale uncertainties.* Treating each observable as an interval rather than a point (the ±20–30% ranges above), all nine central values lie inside their assigned half-rung windows, but eight of the nine intervals reach a window edge, the mean interval half-width being about 0.45 rung, essentially the half-rung tolerance itself. The rung assignments are therefore stable as *central* labels but soft at the edges: the data do not over-determine the ladder.

*(iv) Sensitivity to the time anchor.* Repeating the fit with the anchor $\tau_{0}$ shifted by ±25% (Section 3.1) moves every absolute rung label by $\Delta r=\log_{\varphi }\left(1.25\right)\approx 0.46$ rung, so edge-case labels can change by one integer while the overall fit quality is essentially unchanged. Table 4 reports, for the nine timescales in ascending order, the integer rung labels obtained at $\tau_{0}=60$, 80, and 100 fs together with the mean fractional deviation in each case. The mean deviation is flat at about 11–12% across the anchor range, and the *relative* ordering of the processes across the ladder is unchanged. Only the absolute labels of edge-case entries shift, consistent with the relabelling invariance of Section 2.3.

*(v) Spectral (Rayleigh) test for a preferred spacing.* Checks (i)–(iv) evaluate the ladder for bases chosen in advance and for a discrete list of alternatives. A complementary and more principled question is whether *any* logarithmic spacing is statistically preferred by the data, which is the field-standard way to test for discrete scale invariance: one forms, for each candidate base *b*, the circular phases $\theta_{i}=2\pi \ln\tau_{i}/\ln b$ and evaluates the Rayleigh power $p\left(b\right)=N{\bar{R}}^{2}$, where $\bar{R}$ is the resultant length of the unit phasors and N=9 [49,50]. Under the null of phases distributed uniformly on the circle the single-frequency false-alarm probability is $e^{-p}$, and scanning the band b∈[1.40,1.90] (about nine independent frequencies over the observed 13-rung span) inflates it by the corresponding trials factor. The strongest peak anywhere in the band, at b≈1.53, reaches only p≈2.5, a band-corrected false-alarm probability of about 0.53, so no spacing in the range stands out, and φ sits within this flat landscape ($\bar{R}\approx 0.05$, p≈0.02). With only nine timescales, this flatness reflects the resolving power of the sample rather than a property of any particular base, so the data are consistent with φ without singling it out from its neighbours. This spectral test therefore agrees with the null-model result of check (ii): a set of only nine log-spaced timescales does not, on its own, separate the ladder from a structureless log-spaced spectrum.

Taken together, checks (i)–(v) show that the ladder is a consistent organising scheme that the present nine-timescale set supports: it orders the timescales without over-fitting them, and it provides the scaffold that places the time-domain and frequency-domain observables on one scale. With only nine log-spaced timescales, the data do not, on their own, statistically privilege φ over neighbouring spacings, so the evidential weight of the paper rests on the molecular-dynamics participation law of Section 5, which is measured directly from the vibrational eigenvectors and is independent of the choice of ladder base.

## 4. Cooperative-Transition and Density-Maximum Mapping

This section uses the energy anchor $E_{\mathrm{coh}}$ of Equation (8) to build a calibrated temperature map. The map has two targets: a cooperative-transition temperature $T_{c}$ near the freezing point and a density-maximum temperature $T_{\max}$ near $T_{\mathrm{obs}}=277.15$ K. The resulting values, $T_{c}=271.8$ K and $T_{\max}=277.6$ K, are therefore reported as calibrated matches to $T_{\mathrm{freeze}}=273.15$ K and $T_{\mathrm{obs}}$, not as independent thermodynamic predictions. The section is retained only as a phenomenological extension of the ladder. It is not used as evidence for the spectroscopic bracketing or for the MD participation result.

For $T_{c}$ we use an Arrhenius-style calibration in which the energy scale $E_{\mathrm{coh}}$ is divided by *n* logarithmic ladder steps of size lnφ,

(14) $$T_{c}\;=\;\frac{E_{\mathrm{coh}}}{n\;k_{B}\;\ln\varphi }\;=\;\frac{\varphi^{-5}\;\mathrm{eV}}{8\;k_{B}\;\ln\varphi }\;\approx \;271.8\;K$$

where the integer n=8 is fixed by requiring the formula to reproduce the freezing point. It is a calibration choice, so Equation (14) is a fit to $T_{\mathrm{freeze}}$.

For the density-maximum temperature $T_{\max}$, we adopt a multiplicative shift,

(15) $$T_{\max}\;=\;T_{c}\;\left(1+\varphi^{-8}\right)\;\approx \;277.6\;K$$

which gives the observed density-maximum temperature, which is within about 0.5 K. The exponent −8 is a calibrated shift exponent and is independent of the integer 8 in Equation (14). We do not assign physical meaning to their numerical equality. A microscopic equation-of-state derivation of Equation (15) remains an open problem. Because both the eV assignment for $E_{\mathrm{coh}}$ and the two integer exponents are selected to match near-freezing landmarks, this construction has no standalone predictive power until tested away from bulk ambient water. A change of $E_{\mathrm{coh}}$ over the published H-bond energy range would move $T_{c}$ proportionally, so the numerical agreement should not be read as a derived equation of state. For this reason, the falsifiable observable is the dimensionless offset relation rather than the absolute temperatures: without refitting the shift exponent, a measured $\left(T_{\max}-T_{c}\right)/T_{c}$ outside the broad interval [0.010,0.035] in a modified system would rule out Equation (15) at the precision claimed here.

The choice of electronvolts in Equation (8) is only a unit convention and carries no physical significance. The temperature map is unit-independent. The energy anchor enters $T_{c}$ exclusively through the combination $E_{\mathrm{coh}}/\left(k_{B}\ln\varphi \right)$ of Equation (14), which has the dimension of temperature, so the same $T_{c}$ is obtained in any consistent set of units. In molar units, the anchor is $E_{\mathrm{coh}}=\varphi^{-5}\;\mathrm{eV}\approx 0.0902\;\mathrm{eV}\equiv 8.70\;\mathrm{kJ}\;{\mathrm{mol}}^{-1}$ (using $1\;\mathrm{eV}=96.485\;\mathrm{kJ}\;{\mathrm{mol}}^{-1}$), which lies near the lower end of the documented water–water hydrogen-bond energy range of ∼0–$40\;\mathrm{kJ}\;{\mathrm{mol}}^{-1}$ [44,45,46,47] and is consistent with a single, partially broken hydrogen bond rather than a fully formed one. We adopt the electronvolt form purely because the separate theoretical framework supplies the dimensionless number $\varphi^{-5}$, to which the electronvolt is then attached. Expressing the same anchor as $8.70\;\mathrm{kJ}\;{\mathrm{mol}}^{-1}$ leaves $T_{c}$, $T_{\max}$, and every downstream number in this section unchanged. The physically meaningful statement is therefore not the unit but the magnitude: the cooperative-transition energy scale is a fraction of one hydrogen bond, and it is this per-bond scale, well characterised spectroscopically [47], that the mapping requires.

## 5. Molecular-Dynamics Tests of the Participation Bridge

The model’s participation-number bridge $N_{\mathrm{eff}}\left(n\right)=N\left(n\right)$ admits two distinct empirical readings: (i) a *topological* reading, in which the cumulative Bethe-shell sizes are upper bounds on the breadth-first-search (BFS) reach in the actual H-bond network, and (ii) a *dynamical* reading, in which the same shell sizes are the band-resolved participation ratios $P_{k}$ at the corresponding intermolecular frequencies. Here, BFS is the standard graph traversal that starts from a chosen molecule, and visits all of its directly H-bonded neighbours, then all of *their* not-yet-visited neighbours, and so on shell by shell. The BFS reach at step *n* is the number of distinct molecules reachable within *n* H-bond hops of the starting molecule, which is exactly the network analogue of the cumulative Bethe-shell size N(n). Both readings predict the same band positions, so the rung-bracketing analysis of Section 3 alone cannot discriminate them. We need explicit access to the H-bond graph and to the mass-weighted vibrational eigenvectors of liquid water. We obtain both from TIP4P/Ew molecular dynamics. The simulation setup is described in Section 5.1, the topological test in Section 5.2, and the dynamical test in Section 5.3, and the two are brought together in Section 5.4.

### 5.1. Setup and Simulation Protocol

The molecular-dynamics runs used the TIP4P/Ew rigid four-site potential [51] in OpenMM 8.5 [52], with post-processing in NumPy and SciPy [53,54]. All runs use the same two-stage protocol. First, the box is equilibrated in NPT at 1 atm so that the volume relaxes to the ambient liquid density, ρ≃1.0 g cm^−3^, at the target temperature. Second, the production trajectory is propagated in NVE. This is the appropriate split for vibrational observables: the barostat and thermostat set the state point, but they are removed before measuring low-frequency participation, density of states, and velocity-projection signals, where coordinate rescaling or thermostat friction would perturb the dynamics.

The topological BFS test uses an N=5000 system of rigid water molecules in a cubic periodic box at the ambient liquid density (ρ≃1.0 g cm^−3^), equilibrated at T=298 K (NPT, Monte-Carlo barostat at 1 atm, 100 ps) and then propagated for 250 ps in the NVE ensemble with a Verlet integrator and a 2 fs time step. The dynamical INM participation results reported in the main text use the same NPT-equilibrate/NVE-produce protocol at the ambient density (ρ≃1.0 g cm^−3^) at two temperatures, 298 and 277 K (Section 5.6). Long-range electrostatics used particle-mesh Ewald with an 8.5 Å real-space cutoff. The dynamical INM participation test reported in Table 5 uses two complementary INM analyses. (i) A *translational* analysis: for each of eight instantaneous thermal configurations sampled at ≥20 ps separation along the production trajectory (several times the longest H-bond correlation time of interest, so that they are statistically independent draws), the mass-weighted Hessian of the molecular centre-of-mass translations (three degrees of freedom per molecule) was assembled by finite-difference of the simulation forces and diagonalised numerically, pooling 120,000 modes. (ii) A *rigid-body* 6N cross-check that adds the three molecular rotations per molecule: two configurations were first quenched to a local potential-energy minimum, and the full 6N×6N mass-weighted Hessian diagonalised (60,000 modes). The TIP4P M virtual site, which carries the molecular charge, is included in every force and torque sum, and its position is recomputed after each finite-difference displacement. Because this 6N calculation uses quenched configurations rather than the same eight thermal INM frames, it is treated as a separate delocalisation check, not as statistically identical evidence to the translational-INM analysis. Three numerical checks on the N=5000 box validate the INM calculation: the three acoustic translation modes return to zero (smallest $|\tilde{\nu }|\leq 2.4$ cm^−1^), the density of states peaks at ∼160 cm^−1^ with ≈21% modes of imaginary frequency (negative Hessian eigenvalues) (consistent with the INM literature for water), and the band-averaged participations are stable to ∼5% over a five-fold range of the finite-difference step.

The H-bond adjacency matrix is constructed under two definitions: the distance-only (DIST: r(O,O)<3.5 Å) and the Luzar–Chandler angular criterion (ANG: r(O,O)<3.5 Å and $∠\left(H,O_{d},O_{a}\right)<30^{\circ }$) [55], giving mean coordination ${\langle z\rangle }_{\mathrm{DIST}}=4.73$ and ${\langle z\rangle }_{\mathrm{ANG}}=3.63$, respectively. The latter is the canonical liquid-water value (∼3.5–3.7) and corresponds to a ∼9% broken-bond fraction at 298 K. The two definitions bracket the model assumption z=4. If the Bethe formula is evaluated at the measured mean coordinations rather than at the ideal tetrahedral value, the shell sizes change substantially: for z=3.63, N(1,3,5,6)≈(4.6,39,279,736), while for z=4.73, N(1,3,5,6)≈(5.7,89,1250,4665). These values are not used as replacement predictions, because the Bethe construction is deliberately tied to the ideal tetrahedral valence z=4. They show instead that the measured coordination definition is one source of uncertainty in mapping shell depth to reach. Thus, the Bethe sequence is a strict reach bound only for the ideal local-valence reference. Real-water coordination deficits and ring closure relax that bound and make the shell labels organizational rather than literal MD shell-size predictions.

### 5.2. Topological Test: BFS Shell Sizes in the H-Bond Graph

We measure how far the breadth-first search reaches in *n* steps from a typical molecule in liquid water’s actual H-bond graph, and compare it with the loop-free Bethe value N(n). We compute cumulative BFS shell sizes on (i) two idealised z=4 reference graphs (a diamond-cubic lattice (a regular crystalline z=4 network), and a random graph in which every vertex has exactly four neighbours but the connections are otherwise drawn at random), and (ii) the TIP4P/Ew H-bond network at N=5000, averaged over all 5000 starting vertices and 50 trajectory frames under both the DIST and ANG H-bond definitions of Section 5.1. A depth-7 finite Bethe tree has 4373 vertices and reproduces the ideal cumulative values through shell 7, including shells 5 and 6, so the Bethe-theory row in Table 6 is not affected by finite-tree saturation over the shell range used here.

Table 6 reports the cumulative reach values. Shell 0 is exact by construction. At shells 1 and 2, the MD values agree with Bethe to within ∼17%, with DIST slightly above and ANG slightly below. From shell 3 onward, the MD values fall progressively below Bethe. At shell 5, both definitions are 2.4–3.5× below Bethe. This shortfall is largely a coordination-counting effect rather than a ring effect: the real network has mean coordination ${\langle z\rangle }_{\mathrm{ANG}}\approx 3.63$ instead of the ideal z=4, and re-evaluating the Bethe recurrence at the measured coordination already accounts for most of the reduction through the first few shells. The genuinely loop-related contribution, in which short rings shorten breadth-first-search distances and so reduce the *n*-th reach below the loop-free reference N(n), becomes appreciable only at the larger shells n≳3. Under the canonical ANG definition, the measured reach falls progressively further below the tree, reaching ∼6× below by shell 6. Under the DIST definition, it is marginally above the tree at shells 1–2 (the distance-only criterion over-counts near oxygen neighbours) and ∼4× below by shell 6. The Bethe sequence therefore is retained only as an *upper bound* on topological reach in liquid water, consistent across DIST and ANG. Each finite reference graph in Table 6 saturates at its total vertex count once BFS has visited every node (diamond-cubic and random 4-regular at ∼128). The plateau in those rows at large *n* reflects this finite-size saturation, not any feature of the H-bond network.

The BFS analysis above is purely topological. A genuine band-resolved dynamical test of the participation bridge requires the mass-weighted instantaneous-normal-mode analysis of Section 5.3, presented next.

### 5.3. Dynamical Test: Instantaneous-Normal-Mode Participation

We now test directly whether the band-resolved participation ratio $P_{k}=1/\sum_{i}v_{ki}^{4}$ of the actual mass-weighted vibrational eigenvectors $\left\{v_{k}\right\}$ of liquid water matches the model hierarchy $N_{\mathrm{eff}}\in \left\{5,53,485\right\}$ at the corresponding intermolecular frequencies (180, 60, ≲30 cm^−1^). This is the direct dynamical-participation reading of the bridge, distinct from the topological reading tested in Section 5.2. Note that the three band frequencies (180, 60, ≲30 cm^−1^) are fixed by experiment and by the measured VDOS (Appendix B), not by the ladder, and $P_{k}$ is measured directly on the INM eigenvectors at those frequencies. The $\tau \propto \sqrt{N_{\mathrm{eff}}}$ scaling only labels which shell each band is compared against and does not enter the measured $P_{k}$. So, whether $P_{k}$ agrees with N(n) is an independent empirical comparison.

The test is restricted to the three *odd-numbered* shells (n=1,3,5) because only those map onto separately resolvable far-infrared/THz *bands* of liquid water (Table 2): the H-bond stretch (∼180 cm^−1^), the H-bond bend (∼60 cm^−1^), and the low-frequency connectivity band (≲30 cm^−1^). The even shells 2,4,6 are time-domain ultrafast kinetic processes (vibrational relaxation, H-bond breaking, molecular-jump reorientation) with no isolatable spectral peak, and the INM test (which bins eigenvectors by frequency and averages $\langle P_{k}\rangle$ per window) is meaningful only for distinct, well-separated bands. The three odd shells also span the full range the linear bridge predicts (N=5,53,485, a factor of ∼100), giving maximum discriminating power. Inserting the even shells would add intermediate points without changing the outcome.

Using the translational INM spectrum of Section 5.1 (eight frames of the production N=5000 system), we sorted the modes into the three target frequency bands (180, 60, and ≲30 cm^−1^) and report the average participation ratio $\langle P_{k}\rangle$ in each band. As a basic sanity check, the vibrational density of states peaks near 150–200 cm^−1^, consistent with the published INM literature for liquid water [18,19].

Table 5 compares the measured band-averaged participation ratios with the model values. The measured $\langle P_{k}\rangle$ increase only modestly across the three bands, in contrast to the steep linear sequence 5,53,485: the translational-INM values run from $\langle P_{k}\rangle \approx 677$ at the 180 cm^−1^ band to ≈1317 at the ≲30 cm^−1^ band. The standard errors are small on this scale (Table 5), so even the lowest-frequency band, pooled over 1668 modes, already spreads over more than 485 molecules. A separate rigid-body 6N cross-check that includes molecular rotations gives an even flatter profile (Table 5). In both analyses, the band-to-band variation is at most a factor of two. This is the central measurement of the participation content of the ladder: the low-frequency intermolecular modes are delocalised, so the participation saturates with frequency rather than tracking the shell sizes N(n). The saturating profile is exactly what the delocalisation-saturated law given by Equation (16) describes. The modest variation of $\langle P_{k}\rangle$ also measures the role of the equal-amplitude assumption underlying the $\tau \propto \sqrt{N_{\mathrm{eff}}}$ scaling (Section 2.4.2): most of the shell-to-shell change is carried by a shell-dependent restoring stiffness k(n) rather than by the participation count, so the equal-amplitude reading is a simplification of the measured stiffness behaviour.

The rotation-included 6N Hessian already contains the molecular librations, so binning its modes by frequency probes the most prominent far-IR feature above the translational window (the librational band near 720 cm^−1^). At ambient density, the librational continuum (400–800 cm^−1^) still participates over $\langle P_{k}\rangle \approx 1925$ molecules (≈0.39N), and a finer frequency-resolved decomposition (Appendix C) confirms that participation falls smoothly toward the high-frequency edge but remains delocalised over ∼$10^{3}$ molecules even in the librational band. Frequency-resolving the spectrum therefore confirms the delocalisation directly: even the most localised intermolecular band of liquid water spreads over many molecules, consistent with the saturating participation law established below.

The apparent paradox of comparing N(1)=5 with a measured $P_{k}\approx 677$ is the cleanest statement of the result: the two are different *kinds* of objects. N(1)=5 is an *intensive* topological count (a molecule plus its four neighbours, independent of system size), whereas $P_{k}$ is an *extensive* dynamical quantity that grows with the box (P∼O(N) for delocalised modes, with only the spectral tails localising [19,20]). The linear reading would make the 180 cm^−1^ band a compact five-molecule breathing mode. The data show a *delocalised* network mode spread over ∼0.14N molecules. The meaningful size-independent comparison is therefore the band-to-band participation *ratio*, normalised to the 180 cm^−1^ band, which is invariant under a change of box size.

**Figure 3:**
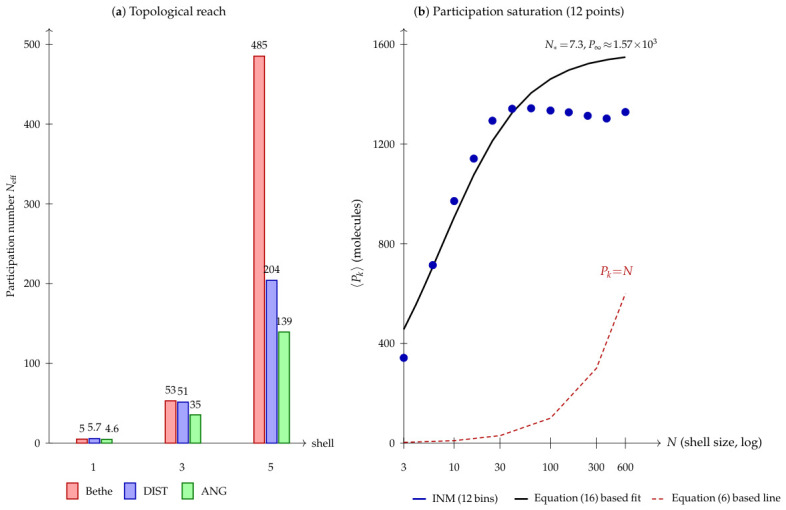
Topology and dynamics of the Bethe-shell participation-number interpretation. (**a**) BFS reach in the actual H-bond graph at shells 1, 3, and 5 under both DIST and ANG H-bond definitions, compared with the Bethe upper bound (red bars). (**b**) The dynamical test as a participation-saturation curve over twelve frequency-resolved points (production N=5000, 298 K, the same data tabulated in Table 7). The band-averaged participation $\langle P_{k}\rangle$ (blue dots) is plotted against the cluster size *N* assigned to each frequency window by the φ-ladder, on a logarithmic *N* axis. The *linear* bridge given by Equation (6) (red dashed, $P_{k}=N$) would have participation track the cluster count. The data instead saturate at a band-limited plateau, and the delocalisation-saturated corrected bridge given by Equation (16) [black curve, joint fit $N_{*}=7.3\approx N\left(1\right)$, $P_{\infty }\approx 1.57\times 10^{3}$], captures the rise-then-plateau shape across the whole band. The participation spans only a factor of ∼4 over the resolvable range, and Table 5 gives the corresponding band-to-band ratios (1:1.93:1.95 measured versus 1:10.6:97 for the linear count).

On this common axis, the participation law is read off directly (Figure 3b). The *linear* reading $N_{\mathrm{eff}}\left(n\right)=N\left(n\right)$ would give a steeply rising profile 1:10.6:97. The translational INM data give a flat 1:1.93:1.95 (from 677:1303:1317) and the rigid-body 6N data 1:1.01:1.01 (from 2379:2404:2404). The measured profile is therefore flat, establishing that participation saturates: the departure from the steep linear profile is much larger than the inter-frame scatter, and it remains clear even under deliberately inflated uncertainty estimates. Thus, the compact linear bridge $N_{\mathrm{eff}}\left(n\right)=N\left(n\right)$ is not validated as the actual participation law of liquid water. This measured saturation is exactly the behaviour the delocalisation-saturated law given by Equation (16) captures. The MD replaces the compact-cluster participation reading with a phenomenological saturated participation law while leaving the empirical frequency placements as a separate organizing step.

Figure 3b shows the same result geometrically. The measured curve starts far above the $P_{k}=N$ line at small assigned shell size ($\langle P_{k}\rangle \approx 342$ at N≈3) and then rises only modestly, reaching ≈1330 at N≈600. The participation law is therefore saturating rather than linear: the absolute values show delocalised modes, and the band-to-band ratios give the size-independent summary of that saturation.

As an independent confirmation, we project the time series of oxygen-atom velocities onto the model’s predicted shell-1 vibrational pattern (the central molecule moving against the mean velocity of its four H-bonded neighbours) and compute the resulting frequency spectrum. The cross-check is now fully specified. For each of the 273 central molecules, we built the shell-1 displacement pattern at the instantaneous H-bond geometry (the root oxygen velocity minus the mean velocity of its four nearest H-bonded neighbours), normalised it to unit Euclidean norm, projected the oxygen-velocity trajectory onto it, and formed the power spectral density (PSD) of the scalar projection. The 273 single-molecule PSDs were then averaged. The unprojected reference is the PSD of the raw oxygen velocities over the same molecules and frames, so the comparison is the dimensionless ratio of integrated band powers, projected over unprojected, in each target window. If the shell-1 pattern were a dynamically distinguished direction, this ratio would exceed unity at 180 cm^−1^. We find no significant amplification: the integrated-power ratio is 1.06 (+5.9%) in the 180 cm^−1^ band (120–240 cm^−1^), 0.99 (−0.8%) at 60 cm^−1^, and 0.88 (−11.7%) below 30 cm^−1^. The noise floor, estimated from the scatter of the same ratio across four control windows between 240 and 720 cm^−1^ (where the shell-1 direction should produce no enhancement), is σ≈4%. The +5.9% enhancement at 180 cm^−1^ is therefore only ≈1.5σ and consistent with zero, while the lower bands show no enhancement at all. Because this projection uses a limited set of central molecules and a simple control-window noise estimate, we treat it only as a supporting null check, not as an independent statistical rejection test. The shell-1 spatial pattern is thus not a special direction of the actual vibrational motion in the H-bond stretch region, in agreement with the INM participation result above.

### 5.4. What the Two Tests Establish

The two tests establish complementary facts about the participation content of the ladder. The *topological* reading is a clean bound: the loop-free Bethe sizes upper-bound the BFS reach of any network of the same valence, and the measured reach $N_{\mathrm{MD}}\left(n\right)$ tracks that bound until ordinary 〈z〉≈3.63 coordination (rather than ring physics) opens a gap (Section 5.2). $N_{\mathrm{MD}}\left(n\right)$ thus describes the connectivity of the network. The *dynamical* reading is the decisive measurement: it shows that participation saturates, with the band-to-band ratio staying within a factor of ∼2 instead of climbing the steep linear 1:10.6:97, and the delocalisation-saturated bridge given by Equation (16) reproduces the frequency-resolved participation curve with a coherence cutoff $N_{*}\approx 7$ (twelve frequency points, Table 7). The shell-1 mode-projection cross-check agrees: there is no preferred-direction amplification, because beyond the first coordination shell, the breathing motion is shared with the surrounding network.

### 5.5. The Corrected Participation Bridge: A Delocalisation-Saturated Law $N_{\mathrm{eff}}=F\left(N\left(n\right)\right)$

The identification $N_{\mathrm{eff}}\left(n\right)=N\left(n\right)$ in Equation (6) is the small-cluster limit: it equates the dynamical participation number with a loop-free topological count, and it holds while a shell-*n* in-phase breathing motion stays coherent over all N(n) molecules. The molecular-dynamics test of Section 5.3 shows that this coherence is limited, so we promote Equation (6) to a bridge that keeps the topological index N(n) as the independent variable but accounts for the finite coherence range of a real network. An in-phase shell vibration can stay coherent only over a limited number of molecules, $N_{*}$. For clusters larger than $N_{*}$, the motion mixes with the many extended vibrations of the surrounding network, so the number of molecules sharing it stops growing with the nominal cluster size and levels off at a plateau $P_{\infty }$ (set by the band and the box size). We use the following Padé-like one-crossover form as the simplest phenomenological interpolation between these limits, not as a unique derivation from first principles:

(16) $$N_{\mathrm{eff}}\left(n\right)\;=\;F\left(N(n)\right)\;=\;\frac{P_{\infty }\;N\left(n\right)}{N\left(n\right)+N_{*}}$$

which interpolates between a *localised* regime, $N_{\mathrm{eff}}≃\left(P_{\infty }/N_{*}\right)\;N\left(n\right)$ when $N\left(n\right)\ll N_{*}$ (linear in the shell count, the original bridge Equation (6), valid only near the first shell), and a *delocalised* regime, $N_{\mathrm{eff}}$ approaches $P_{\infty }$ when $N\left(n\right)\gg N_{*}$ (participation saturates, independent of *n*). The old Equation (6) is the small-cluster tangent of this corrected bridge. To constrain the two parameters, we do not rely on the three named bands alone (a two-parameter law fitted to three points would be weakly determined) but fit the *frequency-resolved* participation curve of the production-run TIP4P/Ew translational INM spectrum (69,241 stable modes from eight N=5000 frames at ambient density ρ≃1.0 g cm^−3^, binned into twelve logarithmically spaced windows from 15 to 225 cm^−1^ and each window centre assigned a continuous shell size *N* through the φ-ladder frequency–rung map; see Table 7). The joint fit over all twelve points gives

(17) $$N_{*}\;\approx \;7.3\;(\mathrm{twelve}\;\mathrm{points};\;\mathrm{three}\text{-}\mathrm{band}\;\mathrm{fit}:\;4.9),\;P_{\infty }\;\approx \;1.6\times 10^{3}$$

Both estimates of $N_{*}$ are of order the first-shell count N(1)=5 (i.e., a few molecules, one coordination shell), which is the physically meaningful statement. The two fits agree on that order of magnitude rather than on a precise value, with a root-mean-square residual of 16% over the twelve points (Table 7). The participation rises from $\langle P_{k}\rangle \approx 342$ at the high-frequency edge to a plateau near 1330, a factor-of-∼4 spread across the resolvable band, not the factor of ∼100 the linear bridge demands. With twelve points, the two-parameter law is over-determined, so the close-but-imperfect fit is a genuine test of the functional form rather than an interpolation through a handful of values. The larger residual relative to a coarse three-band fit (which returns $N_{*}=4.9$ at 3%, of the same order, one coordination shell) is the honest price of that over-determination, and the core, model-independent content is the flat band ratio of Section 5.3, which the fit then summarises as a saturating law. Two features make the fitted parameters physically interpretable. First, the crossover scale comes out at $N_{*}\approx N\left(1\right)$, i.e., *one coordination shell*: coherent shell-breathing survives up to the first neighbour cage and no further, which is exactly where the localised-cluster picture should break down. Second, the rotation-included 6N modes, which couple librations into every band, are even more strongly delocalised and give $N_{*}\;\approx \;0$ (saturated at all shells). We are explicit that $P_{\infty }$ is an *extensive* quantity (≈$0.31\;N_{\mathrm{tot}}$ here) and is therefore calibrated, not predicted. The transferable, system-size-independent content of Equation (16) is the crossover *shape* and the crossover scale $N_{*}$.

The fitted coherence scale $N_{*}\approx 7.3$ has a direct structural interpretation in terms of the solvation environment of a water molecule, and this interpretation is the physical content of the parameter, over and above its role in preventing the unphysical geometric growth of the loop-free shell count. A central water molecule together with its first coordination shell comprises the molecule and its hydrogen-bonded neighbours: the loop-free count places this at N(1)=5 (a molecule plus four neighbours), while integration of the oxygen–oxygen radial distribution function to its first minimum gives a first-shell coordination number of about 4.3–4.7 for the present model [${\langle z\rangle }_{\mathrm{DIST}}=4.73$, ${\langle z\rangle }_{\mathrm{ANG}}=3.63$, Section 5.1], so the central molecule and its complete first shell together contain of order 5–6 molecules. The value $N_{*}\approx 7$ therefore corresponds to coherence that spans the entire first solvation shell and reaches only marginally into the onset of the second. Physically, $N_{*}$ is thus a measured decoherence length rather than a numerical regulariser: a low-frequency intermolecular vibration remains in phase across the first solvation cage of a molecule and then loses coherence as it mixes with the many extended vibrations of the surrounding network. The first solvation shell is, in this precise sense, the natural coherence unit of the low-frequency dynamics of liquid water, consistent with the temperature robustness of $N_{*}$ reported in Section 5.6.

The correction to the participation bridge carries a direct and necessary consequence for the period law, which we now make explicit. The harmonic relation of Section 2.4.2, $\tau \propto \sqrt{N_{\mathrm{eff}}}$ [Equation (13)], is a property of a single collective oscillator and is *not* itself revised. What is revised is the participation number inserted into it. Replacing the small-cluster identification $N_{\mathrm{eff}}\left(n\right)=N\left(n\right)$ by the measured saturating law $N_{\mathrm{eff}}\left(n\right)=F\left(N\left(n\right)\right)$ of Equation (16) turns the period law into

(18) $$\tau \left(n\right)\;\propto \;\sqrt{N_{\mathrm{eff}}\left(n\right)}\;=\;\sqrt{\frac{P_{\infty }\;N\left(n\right)}{N\left(n\right)+N_{*}}}$$

which has two regimes. For $N\left(n\right)\ll N_{*}$, it reduces to the original $\tau \propto \sqrt{N(n)}$, so the ideal-ladder derivation of Section 2.4 survives unchanged near the first coordination shell. For $N\left(n\right)\gg N_{*}$, however, the participation saturates and the period approaches a constant, $\tau_{\mathrm{single}}\sqrt{P_{\infty }}$, so it no longer grows with shell index. Because the measured coherence cutoff is only $N_{*}\approx N\left(1\right)$, essentially all of the resolved low-frequency bands lie in the second, saturated regime. The physically important conclusion is therefore that the corrected participation law does *not* regenerate the geometric rung spacing through $\tau \propto \sqrt{N_{\mathrm{eff}}}$: a ladder whose period grows by φ per rung would require $N_{\mathrm{eff}}$ to keep growing as $\varphi^{2n}$, whereas the measured participation flattens after one shell. This is not an inconsistency but the central physical message stated quantitatively. The near-exponential spacing of the observed timescales is carried by the externally fixed geometric factor φ (the organizing ladder), not by a participation number that grows with cluster size. The saturating law of Equation (16) supplies the participation *content* of each rung, while the rung *spacing* remains an external input. The two are logically independent, and only the small-cluster limit of Equation (18) coincides with the naive $\tau \propto \sqrt{N(n)}$ reading.

The linear bridge $N_{\mathrm{eff}}\left(n\right)=N\left(n\right)$, Equation (6), was first proposed as the mechanism linking network topology to the ladder spacing (from topology to participation to rung spacing, via $\tau \propto \sqrt{N_{\mathrm{eff}}}$). The molecular dynamics do not confirm that mechanism for the actual liquid. They refine this picture: the low-frequency modes are delocalised, so the participation numbers saturate at about one coordination shell rather than growing like N(n), and the MD-calibrated saturating law Equation (16) gives the participation content of each rung directly. The mapping of frequencies onto the ladder is, by contrast, unaffected, and nothing in the MD result bears on it: the rung spacing is set by the external geometric input φ, while the MD now supplies the participation law that populates each rung, so the discrete ladder stands as a calibrated organizing scheme for the band centres.

### 5.6. Robustness: Temperature

Within TIP4P/Ew, the measured participation law is stable over the two temperatures tested. Lowering the temperature from 298 to 277 K (near the experimental density maximum) leaves the translational band-to-band ratio (1:1.86:1.88 versus 1:1.93:1.95) little changed and the twelve-point coherence cutoff barely shifted ($N_{*}=6.6$ versus 7.3, Table 8). The deeper, model-independent reason for this stability (that a vibration spread across a disordered network of masses and springs involves a number of molecules that grows with the size of the sample) is set out in the two paragraphs that follow, which address the system-size and force-field dependence directly.

Two further questions decide how far the participation result generalises: the size of the simulation box and the choice of water model. Enlarging the box changes only one number, and it is not the number we report as the physical result. The plateau participation $P_{\infty }$ is simply a count of how many molecules move together in a single low-frequency vibration. Because these vibrations spread across the whole sample instead of staying trapped in a small cluster, this count grows in proportion to the number of molecules, reaching $P_{\infty }\approx 0.31\;N_{\mathrm{tot}}$ in the present N=5000 system. This growth with system size is expected. It is precisely the signature of a sample-spanning mode, whereas a mode confined to a fixed cluster would give the same $P_{\infty }$ at every box size. The physically transferable content is therefore not $P_{\infty }$ but the two quantities that stay fixed as the box is enlarged: the band-to-band participation ratio and the crossover scale $N_{*}\approx N\left(1\right)$ of one coordination shell. Both are size-independent, and neither is expected to drift once the box is much larger than the coherence length, a condition amply satisfied here, where $N_{*}$ is a few molecules while $N_{\mathrm{tot}}=5000$. Independent studies that vary the system size, on the instantaneous normal modes of liquid water and reaching as large as $1.8\times 10^{4}$ molecules, confirm the same picture: the low-frequency modes are extended, and localisation sets in only in the spectral tails [20]. This supports reporting $P_{\infty }$ as a size-dependent count and the crossover scale $N_{*}$ as the transferable, size-independent content. The same finding is also not tied to the details of any single model. In any disordered network of masses and springs, a vibration that spreads across the sample involves a number of molecules that grows with the size of the sample, so the ratio of participation between neighbouring bands stays close to one instead of climbing as larger shells are assigned to a band. The TIP4P/Ew model is used here because it reproduces the two things the test actually relies on: the shape of the far-infrared/terahertz band and the peak in the density of states near 150–200 cm^−1^. Repeating the instantaneous-normal-mode analysis with a flexible, polarizable, or explicitly many-body potential (for example TIP4P/2005f, the polarizable AMOEBA model [56], MB-pol [57,58,59], or a machine-learned model) is the natural quantitative test of force-field transferability, and is identified as such in Section 6.

There is also a more specific reason to expect the result to carry over to other models. Simple fixed-charge models such as TIP4P/2005 are known to struggle with the far-infrared band near 200 cm^−1^, but the trouble is with how *strongly* that band absorbs infrared light, not with whether the band is there at all. Two fixed charges vibrating against each other barely change the overall dipole of the pair, so matching the measured absorption strength requires adding molecular polarizability or intermolecular charge transfer to the model [60]. The actual *mechanical* motion, the stretching of the hydrogen bond, is still present in the point-charge models regardless of this absorption problem. Because the participation analysis of this paper is built from the mechanical vibration patterns (the eigenvectors of the mass-weighted Hessian) and not from infrared absorption, it measures exactly the part that the different water models agree on. The delocalisation and the participation saturation are therefore expected to be largely unaffected by the electrostatic refinements (polarizability, charge transfer) that most strongly distinguish one model’s spectrum from another, which further supports the transferability of the qualitative result.

## 6. Open Questions and Outlook

Two theoretical problems are left open by the present model. First, the $T_{c}$-to-$T_{\max}$ broadening factor of Equation (15) still needs a microscopic equation-of-state derivation. Second, the residual ring contribution to the BFS reach shortfall would acquire predictive status only if it could be derived independently, for example via a branching reference built from rings rather than single bonds (a “Husimi tree”), which would explicitly include the five- and six-membered rings that dominate liquid water [11].

Three experimental tests remain outstanding. Each is stated below as a single case, together with what it can and cannot establish.

*Test (i): Band-resolved mode participation.* An *experimental* measurement of band-resolved mode participation (for example via isotope-dilution or spatially resolved THz probes) to complement the simulations. This test directly checks the measured participation law: it states a prediction in advance (a compressed, factor-of-a-few participation spread), and the measurement would either confirm or revise the saturating law established in Section 5.3. At the present precision, a normalised band-to-band participation ratio outside roughly (0.5,2.0) would falsify the compressed-ratio version of the law.

*Test (ii): Librational-edge $T_{c}$ reporter.* The temperature dependence of the librational-continuum edge at ∼720–730 cm^−1^ could be tested as a possible reporter of the same near-freezing network change. This is a consistency check: a confirmed $T_{c}$ reporter would be supportive but, given the calibrated status of the thermodynamic mapping (Section 4), could not by itself upgrade that mapping to a prediction.

*Test (iii): Confinement deviation of $T_{\max}-T_{c}$.* A confinement experiment could test whether $\left(T_{\max}-T_{c}\right)/T_{c}$ departs from the bulk-water $\varphi^{-8}\approx 0.02$ ratio of Equation (15) in hydrophobic nanopores. The cleanest version would require an independent estimate that the effective H-bond energy scale $E_{\mathrm{coh}}$ is not strongly shifted by confinement [61]. This is a prediction stated in advance for a future experiment: it specifies a measurable deviation of the $T_{\max}-T_{c}$ relation under confinement, and a null result constrains only that relation, not the ladder.

A fourth, purely computational direction concerns the statistical status of the ladder. The model-selection scan over logarithmic bases and the null-model randomisation of the rung placements, previously listed here as future work, are now carried out in the main text (Section 3.2). They establish that, with only a handful of log-spaced timescales, the bracketing does not statistically single out φ, which is why the paper rests its physical conclusions on the measured participation law. The remaining open computational test is an independent-water-model repeat of the INM participation analysis, which would determine how much of the measured saturation is force-field independent.

A fifth direction concerns systems in which the characteristic timescales are not known in advance, and aqueous solutions in which a solute perturbs the network. Two features of the present framework make both extensions natural. First, the analysis has a descriptive part and a predictive part, and only the descriptive part requires known timescales. The rung bracketing of Section 3 organises *reported* timescales, but the participation measurement of Section 5 is obtained directly from the instantaneous-normal-mode eigenvectors of a molecular-dynamics trajectory and uses no experimental timescale at all except the single anchor $\tau_{0}$. For a hydrogen-bonded liquid whose relaxation times have not been measured (for example an alcohol, an amide, or a deeply supercooled or confined aqueous phase), one can therefore run the same simulation, compute the frequency-resolved participation curve, and *predict* the coherence scale $N_{*}$ and the band-to-band participation ratio. The ladder placement of the resulting bands then becomes a forward prediction rather than a retrospective match, which is a cleaner test of the construction than the bulk-water case treated here. Second, the framework transfers to solutions without modification, because it measures a property of the network rather than of any individual molecule. A solute reorganises the local hydrogen-bond graph (a hydrophobic group depletes and stiffens the first shell, while an ion restructures it through its hydration shell), and the corresponding change in the measured $N_{*}$ and participation ratio is precisely the observable that quantifies how far the solute’s influence on network coherence extends. That solutes measurably alter exactly these collective low-frequency network motions, over ranges that can exceed the first solvation shell, is already established by combined terahertz-absorption and molecular-dynamics studies of solvated sugars and proteins [62,63]. The participation observable proposed here provides a complementary, eigenvector-based measure of the same solute-induced change in network coherence. An INM participation analysis of water around representative hydrophobic and ionic solutes is thus a well-posed and, in our view, informative continuation of the present work. The study reported here establishes the bulk-water baseline against which such perturbations would be measured.

## 7. Conclusions

This work connects three quantities for liquid water within one calibrated golden-ratio construction: the ultrafast and far-infrared/terahertz band placements, the near-freezing temperature map, and the molecular-dynamics participation law. The construction is intentionally presented as a calibrated organizing framework, not as proof that liquid water uniquely selects φ or as a derived equation of state. Three results stand out.

First, the principal hydrogen-bond timescales and the far-IR/THz intermolecular band centres can be placed *together* on one discrete golden-ratio (φ) ladder, anchored to the 80 fs H-bond contraction time and indexed by the loop-free tetrahedral Bethe sequence: each observable falls inside the rung window set by its shell index (Section 3). This paper demonstrates the common placement explicitly: the time-domain ultrafast set and the frequency-domain THz/far-IR set are organized on the same φ-spaced rung scheme from one time anchor, subject to the half-rung windows and anchor uncertainty stated above. A quantitative statistical assessment (Section 3.2) shows that this placement is a consistent ordering that the present data support without statistically singling out φ: a scan over logarithmic bases finds no statistically preferred spacing, φ included, and a null-model test returns a chance probability p≈0.93 that a structureless log-spaced spectrum is bracketed as tightly. The bracketing is therefore reported as organisation, not as evidence that liquid water selects the golden ratio.

Second, the same golden-ratio factor maps the two experimentally observed thermal landmarks of liquid water. With the separate energy calibration stated in Section 4, the ladder recovers both the freezing point and the temperature of maximum density, with the small offset between them following the $\varphi^{-8}\approx 0.02$ rung spacing. One φ-based relation thus links the molecular timescale ladder to the macroscopic density anomaly at the phenomenological level.

Third, and most quantitatively, a TIP4P/Ew instantaneous-normal-mode test measures the participation content associated with the ladder. The measured band-resolved participation follows a delocalisation-saturated law $N_{\mathrm{eff}}\left(n\right)=P_{\infty }\;N\left(n\right)/\left[N\left(n\right)+N_{*}\right]$ with a coherence cutoff $N_{*}$ of about one coordination shell, a result that is consistent across the two tested temperatures, 298 and 277 K. The shell-1 velocity-projection check gives no positive dynamical signature at the assigned 180 cm^−1^ band within its ∼4% noise floor, so the bridge is supported by participation ratios rather than by a distinct velocity-correlation enhancement. This establishes a calibrated, physically interpretable bridge between network topology and vibrational participation, with the φ ladder supplying the rung spacing and the molecular dynamics supplying the participation law that populates it.

## Figures and Tables

**Table 3:** Fit of the nine intermolecular timescales of Table 2 to discrete logarithmic ladders of base *b*, each re-anchored by its own optimal offset. “RMS residual” is the root-mean-square distance to the nearest rung in rung units (max 0.5). The last two columns are the mean and maximum fractional deviation in period. The fit quality varies only weakly across the range, and the spectral test of Section 3.2 shows that none of this variation is statistically significant, so the bracketing singles out neither φ nor any competing base. With only nine timescales, the column-to-column spread of these metrics is within the resampling scatter expected for a set of this size (check (ii)), so the small numerical differences between rows do not define a meaningful ranking of bases.

Base *b*	RMS Residual (Rung)	Mean |Dev|	Max |Dev|
1.400	0.26	7.7%	13.7%
$\sqrt{2}\approx 1.414$	0.24	7.4%	12.7%
1.450	0.20	6.0%	15.1%
1.500	0.23	7.9%	14.6%
φ≈1.618	0.27	11.3%	19.8%
$\sqrt{3}\approx 1.732$	0.20	8.8%	25.0%
1.900	0.17	10.2%	21.6%

**Table 4:** Sensitivity of the rung assignment to the time anchor $\tau_{0}$, varied by ±25% about the nominal 80 fs. Each row lists the integer rung labels of the nine intermolecular timescales of Table 2 (in ascending order of period) and the mean fractional period deviation. Shifting the anchor moves all absolute labels together but leaves the relative ordering and the overall fit quality essentially unchanged, consistent with the relabeling invariance of Section 2.3.

Anchor $\tau_{0}$	Integer Rung Labels (Ascending τ)	Mean |Dev|
60 fs	{6,7,8,10,11,11,13,14,19}	11.2%
80 fs (nominal)	{5,7,8,9,10,10,12,14,18}	11.8%
100 fs	{5,6,7,9,10,10,12,13,18}	11.4%

**Table 5:** Mass-weighted INM participation ratios in the production N=5000 TIP4P/Ew system at ambient density (ρ≃1.0 g cm^−3^, 298 K), compared with the model’s predicted $N_{\mathrm{eff}}$ values. Translational rows: Hessian of the molecular centre-of-mass translations, 8 instantaneous thermal frames (120,000 modes pooled). The three bands are 120–240, 40–90, and 2–30 cm^−1^. The SEM column is the standard error across the 8 independent frames. The final row adds the rotation-included rigid-body 6N cross-check (4 frames, 120,000 modes, band-averaged participations), which is even flatter. The last two columns give the system-size-*independent* comparison: the fractional participation $\langle P_{k}\rangle /N$, and the band-to-band ratio normalised to the 180 cm^−1^ band. The model requires this ratio to climb 1:10.6:97. The data stay within a factor of 2 (translational) or ∼1% (6N), with the modes occupying a roughly band-independent fraction of the whole box.

Band	N_modes_	$\langle P_{k}\rangle$	SEM	Model $N_{\mathrm{eff}}$	$\langle P_{k}\rangle /N$	Ratio/180
180 cm^−1^ (H-bond stretch)	39,936	677	±3.7	5	0.135	1.00
60 cm^−1^ (H-bond bend)	11,389	1303	±3.5	53	0.261	1.93
≲30 cm^−1^ (connectivity)	1668	1317	±13.7	485	0.263	1.95
model prediction	–	5–485	–	5–485	–	1:10.6:97
6N rigid-body (rot.+trans.), band-avg.	120,000	2379–2404	±3–5	5–485	0.48	1.00–1.01

**Table 6:** Cumulative Bethe-tree shell sizes (theory, z=4) versus BFS shell sizes computed from a diamond-cubic lattice, a random 4-regular graph, and an MD simulation of liquid water with two H-bond definitions (DIST and ANG). The measured reach $N_{\mathrm{MD}}\left(n\right)$ falls increasingly below the loop-free z=4 tree as *n* grows. As discussed in the text this shortfall is largely a coordination-counting effect (〈z〉≈3.63 rather than 4).

Shell *n*	0	1	2	3	4	5	6
Bethe theory (z=4, infinite)	1	5	17	53	161	485	1457
Diamond cubic (128 vertices)	1	5	17	41	77	109	123
Random 4-regular (128 vertices)	1	5	16	45	96	127	128
TIP4P/Ew DIST	1.0 ± 0.0	5.7 ± 0.0	19.8 ± 0.1	51.4 ± 0.3	109.7 ± 0.6	204.0 ± 1.1	343.7 ± 1.8
TIP4P/Ew ANG	1.0 ± 0.0	4.6 ± 0.0	14.3 ± 0.1	35.4 ± 0.2	74.7 ± 0.4	139.3 ± 0.8	236.5 ± 1.4

**Table 7:** Frequency-resolved test of the delocalisation-saturated bridge, Equation (16). The pooled N=5000 TIP4P/Ew translational INM spectrum (ambient density, 298 K, eight frames) is binned into twelve logarithmically spaced windows: $\tilde{\nu }$ is the window centre, *N* the continuous Bethe-shell size assigned by the φ-ladder frequency–rung map, $\langle P_{k}\rangle$ the band-averaged participation, and F(N) the corrected-bridge value at the joint best fit $N_{*}=7.3$, $P_{\infty }=1.57\times 10^{3}$ (rms 16% over all twelve points).

$\tilde{\nu }$ (cm^−1^)	*N*	$\langle P_{k}\rangle$	F(N)
14.7	600	1328	1548
18.9	383	1302	1538
24.2	244	1313	1521
31.0	155	1327	1497
39.7	99	1334	1459
50.9	63	1343	1403
65.2	40	1341	1323
83.5	25	1293	1212
107	16	1141	1066
137	10	971	888
176	6	714	690
225	3	342	488

**Table 8:** Temperature robustness of the translational INM participation result for TIP4P/Ew at ambient density (N=5000, constant-pressure equilibration). Columns give the band-averaged participation ratios, the band-to-band ratio normalised to the 180 cm^−1^ band, the coherence cutoff $N_{*}$ from the twelve-point corrected-bridge fit [Equation (16)], and the residual.

Run	${\langle P\rangle }_{180}$	${\langle P\rangle }_{60}$	${\langle P\rangle }_{<30}$	Ratio (Norm. 180)	$N_{*}$	rms %
298 K (production)	677	1303	1317	1:1.93:1.95	7.3	16.0
277 K	712	1326	1339	1:1.86:1.88	6.6	10.9
linear bridge N(n)	5	53	485	1:10.6:97	–	–

## Data Availability

The data supporting the reported results (the OpenMM 8.5 simulation input files, the analysis scripts that compute the BFS shell sizes and the mass-weighted instantaneous-normal-mode participation ratios, and the figure-generation scripts) are available from the corresponding author upon reasonable request.

## Appendix A. Intramolecular Vibrations: Rung Placements

The intramolecular O–H stretch (∼10 fs) and H–O–H bend (∼1640 cm^−1^, ∼20 fs) are not shell-*n* network modes of the Bethe construction, because they involve no H-bond network topology. They are excluded from the ladder-performance claims. Here, we only record where they would fall if placed on the same φ-ladder. These placements are bookkeeping only and are not counted as evidence for the network ladder.

For the O–H stretch, rung 1 (∼10 fs) is not a Bethe-shell vibration. The $\varphi^{\;8}$ that appears here is a *stiffness* ratio, not a rung index, and follows in two steps. (1) Rung 1 sits four rungs below the rung-5 anchor, and each rung is one factor of φ in period, $\tau_{r}=\tau_{5}\;\varphi^{\;r-5}$, so $\tau_{1}=80\;\mathrm{fs}\times \varphi^{-4}\approx 11$ fs (the ∼3400 cm^−1^ stretch) and the rung-5-to-rung-1 period ratio is $\varphi^{4}$. (2) In the harmonic model of Section 2.4.2, at fixed effective mass $\omega =\sqrt{k/\mu }\propto 1/\tau$, so stiffness scales as the square of frequency: $k_{1}/k_{5}={\left(\tau_{5}/\tau_{1}\right)}^{2}={\left(\varphi^{4}\right)}^{2}=\varphi^{8}\approx 47$. This covalent-to-H-bond stiffness ratio falls inside the published range ∼15–90 [61,64]. Because the range is broad and the mode is intramolecular, this comparison is not used as a ladder-performance check.

The H–O–H bend is the other intramolecular placement recorded here. It sits near the unused rung 2 and gives

(A1) $$\tau_{\mathrm{bend}}\;=\;\tau_{0}\;\varphi^{2}\;\approx \;18.9\;\mathrm{fs},\;{\tilde{\nu }}_{\mathrm{bend}}\;\approx \;1766\;{\mathrm{cm}}^{-1}$$

The obtained bend value is within about 8% of the observed liquid-water bend at ∼1640 cm^−1^ [12]. This is recorded only as a placement on the same ladder, not as evidence for a Bethe-shell network mode.

## Appendix B. Vibrational Density of States of TIP4P/Ew Water

The participation-bridge tests of Section 5 assign the shell-1 band to the experimentally observed ∼180 cm^−1^ H-bond stretch [7,9,10], so we check whether the TIP4P/Ew oxygen-velocity spectrum contains a corresponding VDOS feature. A separate fine-cadence trajectory (same force field, N=5000, and protocol as Section 5.1, with velocities written every 4 fs over 50 ps of NVE production) gives the bulk VDOS as the power spectrum of the oxygen-velocity autocorrelation, averaged over all 5000 oxygens.

In the 150–220 cm^−1^ window, the simulated VDOS has two maxima, at 183 and 207 cm^−1^, both inside the rung-6–7 window of Table 2: the 183 cm^−1^ peak aligns in frequency with the experimental H-bond-stretch band, while the 207 cm^−1^ peak is a TIP4P/Ew-specific feature. The simulation therefore contains the shell-1 spectral feature used in the tests of Section 5.2 and Section 5.3. The 20 ps record resolves ∼1.7 cm^−1^, finer than the ∼5 cm^−1^ structure of interest, and applying a Hann window shifts the 183 cm^−1^ peak by <2 cm^−1^ without changing the assignment.

## Appendix C. Frequency-Resolved Rigid-Body (6N) Spectrum

Frequency-resolving the rigid-body 6N spectrum shows the libration–translation structure explicitly: participation falls smoothly from $\langle P_{k}\rangle \approx 2370$ (0.47N) in the 0–150 cm^−1^ translational region to ≈1845 (0.37N) at 500–700 cm^−1^ and ≈1064 (0.21N) in the librational band (700–760 cm^−1^), continuing to drop toward the high-frequency edge as expected for instantaneous-normal-mode localisation in the spectral tails [19,20].
